# Preparation and Characterization of Cumin Essential Oil Nanoemulsion (CEONE) as an Antibacterial Agent and Growth Promoter in Broilers: A Study on Efficacy, Safety, and Health Impact

**DOI:** 10.3390/ani14192860

**Published:** 2024-10-04

**Authors:** Muhammad Jabbar, Irfan Baboo, Hamid Majeed, Zahid Farooq, Valiollah Palangi, Maximilian Lackner

**Affiliations:** 1 Department of Zoology, Cholistan University of Veterinary and Animal Sciences (CUVAS), Bahawalpur 63100, Pakistan; zoologistofficial@gmail.com (M.J.); zahidfarooq@cuvas.edu.pk (Z.F.); 2 Department of Food Science and Technology, Cholistan University of Veterinary and Animal Sciences (CUVAS), Bahawalpur 63100, Pakistan; hamidmajeed@cuvas.edu.pk; 3 Department of Animal Science, Faculty of Agriculture, Ege University, 35100 Izmir, Türkiye; valiollah.palangi@ege.edu.tr; 4 Department of Industrial Engineering, University of Applied Sciences Technikum Wien, 17 Hoechstaedtplatz 6, 1200 Vienna, Austria

**Keywords:** essential oil, nanoemulsion, chicken, cuminaldehyde

## Abstract

**Simple Summary:**

Broiler meat is widely consumed around the globe. To fulfill consumer demand, broiler farmers are using antibiotic growth promoters for improved efficiency. However, these antibiotics have been banned in developed countries due to antimicrobial resistance. Therefore, plant supplements in the form of essential oils (EOs) have gained acceptability due to their excellent antibacterial properties. The next step is to encapsulate the EO for better delivery to target organs in broilers, which was investigated in vivo. The novelty of this study is to encapsulate volatile compounds of EOs in nanoemulsion (NE). This study has observed a positive effect of NE in terms of broiler growth performance, suggesting that cumin EO NE (CEONE) can be used as a substitute for the prophylactic administration of synthetic antibiotics.

**Abstract:**

This research characterized and explored the effect of cumin essential oil nanoemulsion (CEONE) on broiler growth performance, serum biochemistry, hematological parameters, and cecal microbial count. Day-old (*n* = 96) broilers (Ross 308) were randomly assigned to six treatments with five replicates of three broilers each. The dietary treatments consisted of negative control (only basal diet), positive control (basal diet + 200 µL of enrofloxacin), 25 µL (basal diet + 25 µL of CEONE), 50 µL (basal diet + 50 µL of CEONE), 75 µL (basal diet + 75 µL of CEONE), and 100 µL (basal diet + 100 µL of CEONE). The broiler’s body weight gain (BWG) after 42 days of treatment exhibited increased weight in the CEONE group (976.47 ± 11.82–1116.22 ± 29.04). The gain in weight was further evidenced by the beneficial microbe load (10^7^ log) compared to the pathogenic strain. All the biochemical parameters were observed in the normal range, except for a higher level of HDL and a lower LDL value. This safety has been validated by pKCSM toxicity analysis showing a safe and highly tolerable dose of cuminaldehyde. In conclusion, this research observed the potential of CEONE as a multifunctional agent. It is a valuable candidate for further application in combating bacterial infections and enhancing animal health and growth.

## 1. Introduction

The global demand for broiler meat has surged due to population growth and dietary preferences. Broiler farming plays a crucial role in fulfilling the demand for affordable animal protein [1]. However, to ensure sustainable growth, the poultry sector must focus on improving growth rates, feed efficiency, and bird health. Traditionally, broilers were treated with antibiotics to enhance growth, manage infections, and to maintain gut health [2]. Unfortunately, the extensive use of synthetic AGPs has led to antibiotic resistance, posing environmental and public health risks [3,4]. Many countries have banned such prophylactic antibiotic administrations to tackle antimicrobial resistance. As a result, researchers are now exploring alternatives to achieve high broiler production.

Phytobiotics have been used as feed additives in animal nutrition. EOs, in particular, have garnered attention for their growth-promoting and antimicrobial properties. EOs have been used in the broiler industry to improve digestive enzyme activity and to promote the production of intestinal mucus [5]. Among these natural alternatives, cumin (*Cuminum cyminum* L.) is gaining much attention due to flavor spice [6]. These volatile substances offer various benefits, including antibacterial, antioxidant, anti-inflammatory, and immunomodulatory properties [7]. Cumin enhances broiler health and growth performance by preventing microbial pathogens and increasing the beneficial gut flora [8].

Nanotechnology holds immense promise for broiler production due to the increased bioavailability and efficacy of bioactive compounds [9]. Nanotechnology operates at incredibly small scales, typically ranging from 1 to 100 nanometers. At this level, scientists manipulate and engineer materials with precision [10]. The application of nanominerals, nanocapsules, nanoparticles, and nanoemulsions as feed additives has demonstrated significant potential in promoting growth, immunity, and microbial pathogen avoidance. Their small size, high surface area, excellent homogeneity, and physical reactivity contribute to improved bioavailability and efficacy in livestock and poultry [11]. Numerous studies have explored the use of nanominerals, which are nanoparticles from inorganic compounds, as alternatives to antibiotics, demonstrating reduced feed costs and enhanced growth performance [12,13]. A nanocapsule is a type of hollow core–shell particle designed to encapsulate various substances, providing protection against degradation and enabling controlled release [11]. Encapsulating extracts from graviola leaves [14], garlic [15], peppermint [16], basil oil [17], and CEO [5] in nanocapsules enhances their antimicrobial and antioxidant properties, thereby optimizing growth, gastrointestinal health, and economic efficiency in livestock and poultry. Additionally, probiotics contained in nanocapsules have shown improved growth and gut health [18]. Administering vitamin C nanoparticles with nanomineral zinc oxide reduces heat stress in broiler chickens, improving growth, immune response, intestinal health, and blood parameters [19]. NEs are colloidal dispersions of immiscible liquid droplets, stabilized by surfactants. Kinetic stability, optical transparency, high surface area, and tunable rheology are considered as unique properties of NEs. These NEs enhance the absorption of lipophilic compounds, such as vitamins, EOs, and fatty acids, in the gastrointestinal tract of animals [20]. NEs enable the efficient encapsulation of active compounds by protecting them from environmental factors with controlled-release characteristics [11]. The growth performance effects of NEs have been studied by many scientists using eugenol, thymol, peppermint, and coriander [21,22,23,24].

The aim of this study was to prepare and characterize the CEONE for the identification of the droplet size, PDI, and zeta potential. In this study, comparative oral bioavailability characterization and optimization of antibacterial properties by the molecular docking of organic and synthetic compounds with a Stitch-based interactive protein was performed. Further in vitro validation of the antibacterial activity of the CEONE and in vivo experiments were performed to compare the effect of organic and synthetic compounds on the broilers’ growth, cecal microbial load, blood hematology, and serum biochemistry.

## 2. Materials and Methods

CEO, with a purity of 99%, was purchased from Karachi essence House, Lahore, Pakistan. The reagents Tween 80, De Man–Rogosa–Sharpe (MRS), and total plate count (TPC) agar were purchased from Sigma Aldrich, St. Louis, MO, USA. Day-old chicks and their poultry feed were purchased from Faisal chicks & feeds Pvt. Ltd., Multan, Pakistan. Chemicals used in this study were of analytical grade.

### 2.1. Ethics Approval

The protocol (ORIC-255) was approved by the experimental animal ethics committee of Cholistan University of Veterinary and Animal Sciences (CUVAS). The study was conducted in the laboratory of livestock nutrition at the department of zoology, Cholistan University of Veterinary and Animal Sciences, Pakistan.

### 2.2. Preparation of CEONE

The formulations were composed of CEO as the oil phase and Tween 80 as the surfactant. Tween 80 (CAS no. 9005-65-6) is a polysorbate. CEONE was prepared using a probe ultrasonicator (UCD-1200, BIOBASE, Jinan, China). The 5–15 mL CEO oil phase was mixed with 2–3 g Tween 80 surfactant, followed by a 10–20 min sonication interval for coarse emulsion preparation using DH-1500 inverted homogenizer at 18,000 rpm. Prepared NE was further characterized for droplet diameter, PDI, and zeta potential using a Zeta-Sizer (3000 HS, Malvern Instruments, Malvern, UK).

### 2.3. Transmission Electron Microscopy (TEM)

The morphology of CEONE droplets was investigated through digital imaging using a transmission electron microscope (TEM) (JEOL 2100, Hitachi High-Technologies Corp., Tokyo, Japan). A droplet of CEONE was placed on a carbon-coated copper grid with a dropper and stained with 2% (*w*/*v*) phosphotungstic acid. The carbon-coated copper grid was dried for 24 h before analysis. The examination was conducted at an accelerating voltage of 100 kV [25].

### 2.4. Gas Chromatography and Mass Spectroscopy (GC-MS) Analysis

GC-MS analysis was performed according to Kelidari et al. [26], using a set of 7890A Network GC system coupled with a 5975C VL MSD with Triple-Axis Detector (Agilent Technologies, Santa Clara, CA, USA). An HP-5MS silica-fused column was used for the separation of components, with 40 °C as the initial temperature for 1 min, followed by a 3 °C/minute increase in temperature of up to 250 °C as the final temperature for 90 min. Split flow (10 mL/min with 6 mL/min septum purge, and 1 mL/min column flow rate) was applied and helium was used as a carrier gas with a purity of 99.99%.

### 2.5. In Silico Oral Bioavailability Characterization and Molecular Docking

Cuminaldehyde, being the main constituent of CEO (CAS no. 122-03-2), was compared with enrofloxacin (CAS no. 93106-60-6) as a synthetic antibacterial compound using Swiss ADME (ADME stands for absorption, distribution, metabolism, and excretion. These are the four key processes that describe how a drug or compound behaves in the body). This method has been previously utilized for drug characterization by notable researchers [27,28]. The toxicity assessment was performed using four databases (Toxicity by ADMET Predictor, ProTox 3.0, Toxicity Estimation Software Tool (TEST version 5.1.2) and pKCSM) to achieve conclusive and reliable data. All four databases predicted similar toxicity levels. Therefore, only the predicted data of one database pKCSM (https://biosig.lab.uq.edu.au/pkcsm/prediction accessed on 20 March 2024) have been presented [29,30]. The Stitch database (http://stitch.embl.de/ accessed on 20 March 2024) was used for ligand–protein and protein–protein interaction by entering the ligand name (cuminaldehyde and enrofloxacin) into the search bar while *Escherichia coli* K12 MG1655 was selected as the organism [31]. Interacting proteins showing 100% similarity were selected for further analysis, and the validation of these selected proteins was performed by Ramachandran Plot using PROCHECK analysis [32]. All the chemical structures were retrieved from Pubchem database (https://pubchem.ncbi.nlm.nih.gov/ accessed on 20 March 2024) [33]. Uniport system (https://www.uniprot.org accessed on 20 March 2024) was used to collect the function data of the concerned protein. The interacting protein structures were retrieved by the Protein Data Bank system (https://www.rcsb.org/ accessed on 20 March 2024) [34]. Molecular docking was conducted using the SwissDock tool of the Swiss institute of bioinformatics (http://www.swissdock.ch/ accessed on 22 March 2024) and the ligand with the lowest binding affinity was considered for favorable interaction with the docking receptor [35]. All the docking results were visualized by UCSF chimera version 1.17.3 (3D and solid surface) [36], while the 2D structure was obtained using BIOVIA Discovery Studio Visualizer (version 4.5) [37].

### 2.6. Minimum Inhibitory Concentration (MIC) and Time–Kill Dynamics of CEONE

A liquid culture test was conducted to determine the Minimum Inhibitory Concentration (MIC) of CEONE. Different concentrations of CEONE (80 µL, 100 µL, 120 µL, 150 µL, and 200 µL) were added to 100 µL of nutrient broth and 10 µL of bacterial cultures (*E. coli* and *S. aureus*) in a 96-well microtiter plate. The mixtures were incubated for 24 h, and the optical density (OD) was measured at 600 nm. Time–kill dynamics were observed at 48 and 72 h [38].

### 2.7. Animals, Diets, and Formulations

In this experiment, 90-day-old male Ross 308 broiler chicks were randomly assigned to 6 treatment groups, with 5 replicates of 3 broilers each. All the birds were properly acclimatized, and then grouped as a negative control group (only basal diet), positive control (basal diet + 200 µL of enrofloxacin), 25 µL (basal diet + 25 µL of CEONE), 50 µL (basal diet + 50 µL of CEONE), 75 µL (basal diet + 75 µL of CEONE), and 100 µL (basal diet + 100 µL of CEONE) and treatments were provided during the growth period (days 15–28) (Table 1). During the project, animal welfare was the main goal, which has been strictly followed according to the ethical review committee guidelines from CUVAS (ORIC-255).

### 2.8. Growth Performance

On day 14, the initial body weight (BW) was recorded. After treatments, BW, body weight gain (BWG), feed intake (FI), and feed conversion ratio (FCR) were recorded at 21, 28, and 42 days of age.

FCR = FI/BWG(1)

The mortality rates were assessed daily, and any deceased birds were promptly sent to the pathology laboratory for further analysis. The feed was then adjusted accordingly.

### 2.9. Blood Hematology and Serum Biochemistry

Blood was collected from the wing vein by a syringe containing heparin. Red blood cells (RBCs), white blood cells (WBCs), hemoglobin (Hb), lymphocytes, and platelets were measured by a Mindray BC-1800 hematology analyzer (Mindray, Shenzhen, China). Biochemical parameters were evaluated by a CONTEC BC300 Semiautomatic Blood Biochemistry Analyzer (Contec Medical Systems, Qinhuangdao, China).

### 2.10. Microbial Analysis

On day 42, cecal contents were collected aseptically and stored at 4 °C in a sterile container. One gram of cecal content was diluted in a 10-fold serial solution with PBS, and five g was used for plating. MRS agar was used to culture *Lactobacillus* species, while the total coliform count was determined using TPC agar. *Lactobacillus* plates were counted after 48 h of inoculation, and total coliform plates were counted after 24 h at 37 °C [39].

### 2.11. Statistical Analysis

One-way ANOVA in IBM SPSS Statistics, version 22, was used for statistical data analysis and Tukey’s test was applied for mean separation. Results were represented as means and standard deviation with *p*-value −0.05. For the optimization, the Box–Behnken design of response surface methodology was used (Design Expert version 13 software). The sonication time (min), concentration of Tween 80 (g), and EO (ml) were factor variables while size (nm), PDI (nm), and zeta potential (mV) were dependent variables.

## 3. Results

### 3.1. Chemical Composition of CEO by GCMS

**Figure 1:**
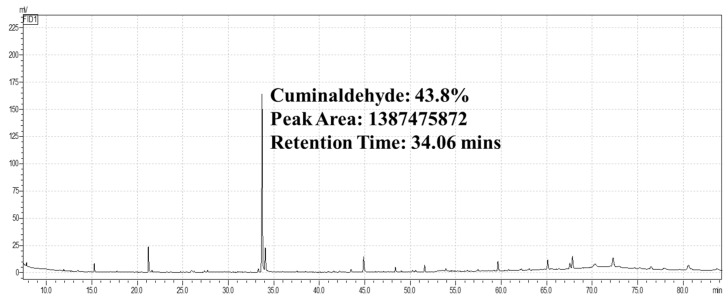
GCMS graph of CEO with maximum peak area: horizontal X-axis showing retention time and vertical *Y*-axis showing peak area for determination of the major compound.

In the GCMS analysis, compounds with a higher concentration were considered. The highest peak area was observed for cuminaldehyde (43.81%) at a retention time of 34.06 min (Figure 1).

### 3.2. Characterization of CEONE

#### Mean Droplet Size, PDI, and Zeta Potential

The following regression equation models the relationship between the mean droplet size (Y) and three independent variables (X_1_, X_2_, and X_3_):

Y = − 0.003293 + 0.044262X_1_ + 0.002679X_2_ + 0.005647X_3_ + 0.000090X_1_X_2_ − 0.000158X_1_X_3_ − 0.000062X_2_X_3_ − 0.008302X_1_^2^ − 0.000122 X_2_^2^ − 0.000142X_3_^2^ (2)

**Figure 2:**
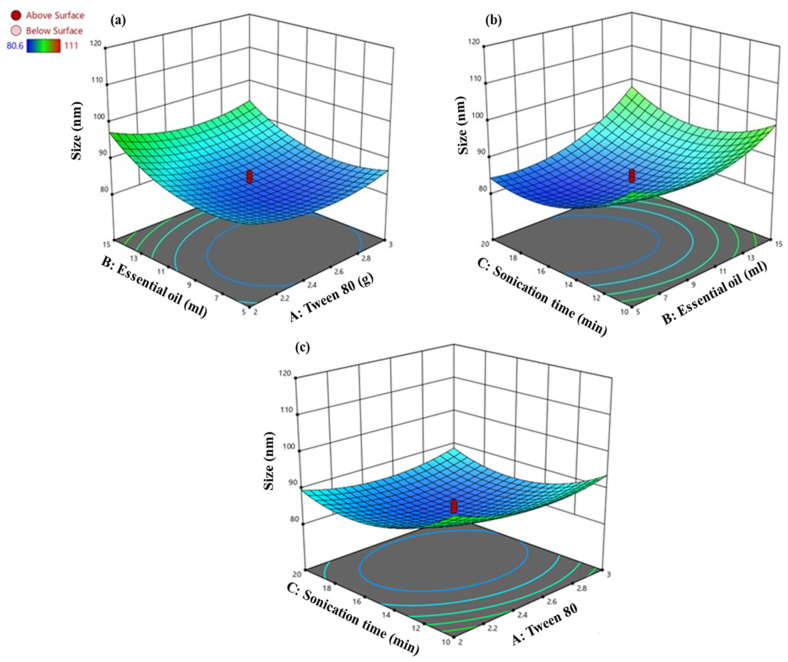
Interaction between (**a**) EO and Tween 80, (**b**) sonication time and EO, (**c**) sonication time and Tween 80 on droplet size of CEONE. The red circles represent the central points.

NE optimization via BBD provided an intercept value of (−0.003293), which indicated the expected droplet size when the Tween 80 concentration, EO volume, and sonication time are at their central levels. The linear coefficients revealed an increase in the Tween 80 (+0.044262X_1_), EO volume (+0.002679X_2_), and sonication time (+0.005647X_3_) that contributes to an increase in the droplet size. The interaction effects showed a nuanced relationship; while the combined effect of the Tween 80 and EO volume (+0.000090X_1_X_2_) slightly increases the droplet size, the interaction between the Tween 80 and sonication time (−0.000158X_1_X_3_) decreases it, and the interaction between the EO volume and sonication time (+0.000062X_2_X_3_) again increases it. The quadratic terms suggest an optimal concentration for the Tween 80 (−0.008302X_1_^2^) and EO volume (−0.000122X_2_^2^), as well as an optimal sonication time (−0.000142X_3_^2^), where the droplet size initially increases, which later drops at a certain point. The graphical analysis in Figure 2 further elucidates these relationships, showing that at a low EO volume, an increase in the Tween 80 concentration initially decreases the droplet size, which then increases beyond a certain point. The smallest droplet size, approximately 85 nm, is observed at 12 mL EO and 2.5 g Tween 80. Additionally, an extended sonication time significantly reduces the droplet size, which suggests the critical impact of sonication on droplet size modulation.

Y = +0.837674 − 0.297612X_1_ + 0.009504X_2_ − 0.043002X_3_ − 0.004945X_1_X_2_ + 0.000192X_1_X_3_ − 0.000418X_2_X_3_ + 0.065673X_1_^2^ + 0.000575X_2_^2^ + 0.001408X_3_^2^ (3)

**Figure 3:**
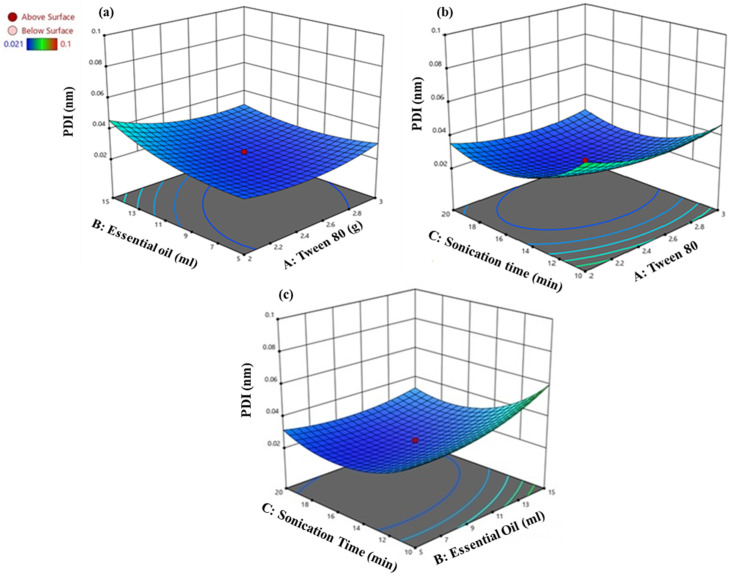
Interaction between (**a**) EO and Tween 80, (**b**) sonication time and Tween 80, (**c**) sonication time and EO on PDI of CEONE. The red circles represent the central points.

The intercept for PDI has been presented as +0.837674. Increasing the Tween 80 concentration (−0.297612X_1_) decreased the PDI, indicating a more uniform droplet size, while increasing the EO volume (+0.009504X_2_) slightly raised the PDI, suggesting less uniformity. The sonication time (−0.043002X_3_) also reduces the PDI, implying improved uniformity. The interaction terms reveal a combined increase in the Tween 80 and EO (−0.004945X_1_X_2_) that significantly decreases the PDI, whereas the interaction of the Tween 80 and sonication time (+0.000192X_1_X_3_) slightly increases it. However, the EO with sonication time (−0.000418X_2_X_3_) decreases the PDI, indicating better uniformity. The quadratic terms showed a nonlinear relationship; the PDI initially decreased with the increase in the Tween 80 (+0.065673X_1_^2^), EO volume (+0.000575X_2_^2^), and sonication time (+0.001408 X_3_^2^). Figure 3 indicates a narrow PDI at 5 mL EO and 2 g Tween 80. The notable decrease in the PDI with the increased sonication time, particularly at 15 min and 2.5 g Tween 80, highlights the critical role of the sonication time in achieving a uniform NE droplet size.

Y = +18.09837 − 10.89017X_1_ − 1.03451X_2_ − 2.09516X_3_ − 0.196500X_1_X_2_ + 0.088500X_1_X_3_+ 0.009150X_2_X_3_ + 2.14479X_1_^2^ + 0.075188X_2_^2^ + 0.052561X_3_^2^ (4)

**Figure 4:**
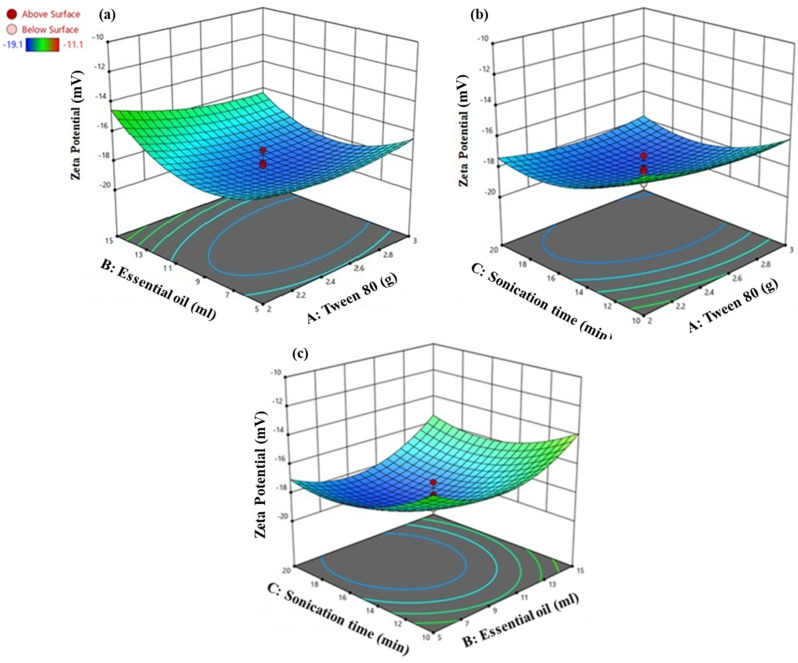
Interaction between (**a**) EO and Tween 80, (**b**) sonication time and Tween 80, (**c**) sonication time and EO on zeta potential of CEONE. The red circles represent the central points.

The BBD suggests an intercept of +18.09837 for the zeta potential. The zeta potential was decreased with concentrations of Tween 80 (−10.89017X_1_) and EO (−1.03451X_2_). On the other hand, the sonication time (−2.09516X_3_) indicates a more negative zeta potential. The interaction terms revealed the combined effects of the Tween 80 and EO (0.196500X_1_X_2_) that significantly decreased the zeta potential. Moreover, the interaction between the Tween 80, sonication time (+0.088500 X_1_X_3_), and EO with the sonication time (+0.009150X_2_X_3_) slightly increased it. The quadratic terms indicate a nonlinear relationship of the zeta potential with the Tween 80 (+2.14479X_1_^2^), EO volume (+0.075188X_2_^2^), and sonication time (+0.052561X_3_^2^). The optimized zeta potential value was achieved at a specific concentration and sonication time (12 mL EO and 2.5 g Tween 80 (−18.5 mV). These results suggest a delicate balance is required in the formulation parameters to maintain the electrostatic stability of the NE (Figure 4).

### 3.3. Morphology Visualization of CEONE by TEM

**Figure 5:**
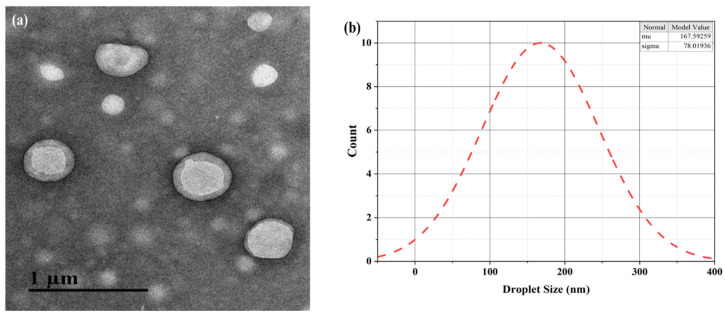
(**a**) TEM micrograph image of CEONE: (**b**) normal distribution model of droplet size.

The morphology of CEONE was visualized by TEM, as shown in Figure 5a,b. The TEM visualization has confirmed a 20–200 nm droplet-sized NE. The droplet size obtained via TEM was 167.59 ± 78.01 nm.

### 3.4. In Silico Analysis

#### 3.4.1. Drugs Characterization

**Figure 6:**
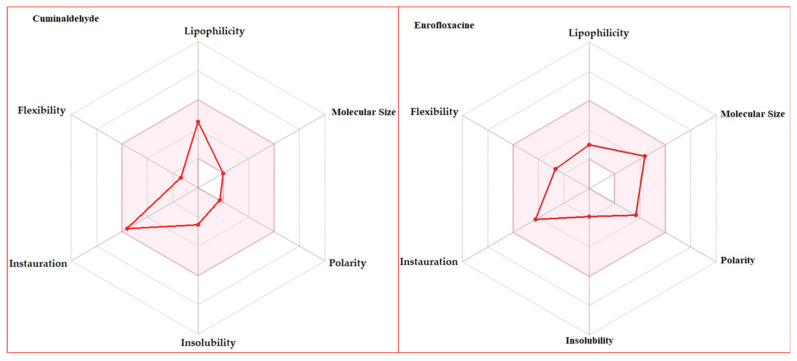
Oral bioavailability radar plot of cuminaldehyde and enrofloxacin.

The oral bioavailability radar graph of cuminaldehyde and enrofloxacin existence in the pink area suggests an optimal range (Figure 6).

According to the Swiss ADME parameters, the MWs of cuminaldehyde and enrofloxacin were 359.39 (g/mol) and 148.2 (g/mol), respectively. Enrofloxacin and cuminaldehyde have Log P_o/w_ values of 1.64 and 2.48, respectively. The TPSA values for enrofloxacin and cuminaldehyde were 65.78 Å² and 17.07 Å², respectively (Table 2). The synthetic accessibility score for enrofloxacin was 2.73, while cuminaldehyde had a score of 1. The oral bioavailability score was similar for both drugs, i.e., 0.55 (Supplementary Tables S1–S5).

The toxicity analysis revealed neither compound exhibited AMES toxicity. The maximum tolerated dose for enrofloxacin was 0.51 (log mg/kg/day), while cuminaldehyde had 0.839 (log mg/kg/day). The LD_50_ values were observed as 1.7 mM for cuminaldehyde and 2.431 mM for enrofloxacin. The hepatotoxicity predictions for enrofloxacin (2.408 log mM) and cuminaldehyde (0.819 log mM) suggest safer limits (Table 3).

#### 3.4.2. Ligand–Protein and Protein–Protein Interaction

**Figure 7:**
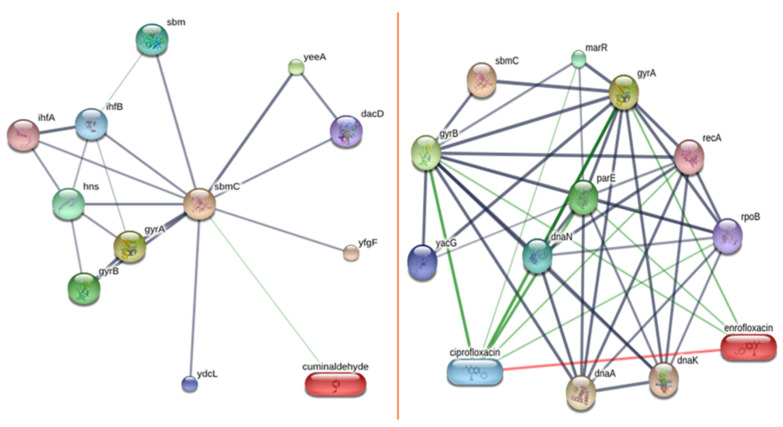
Interaction of cuminaldehyde and enrofloxacin with sbmc.

According to the drug protein interaction data, cuminaldehyde directly interacts with sbmc, while enrofloxacin interacts with gyrA and gyrB. Sbmc with 100% identity and its associated protein (PDB: 1jyh; DNA gyrase inhibitor) showed purity. Molecular docking studies between *Staphylococcus aureus*, enrofloxacin, and cuminaldehyde were not performed due to the lack of interacting protein model data in the Stitch database (http://stitch.embl.de/ accessed on 20 March 2024) (Figure 7).

The Ramachandran Plot statistics showed a quality protein with 94% residues in the allowed region (Supplementary Figure S1).

#### 3.4.3. Molecular Docking

**Figure 8:**
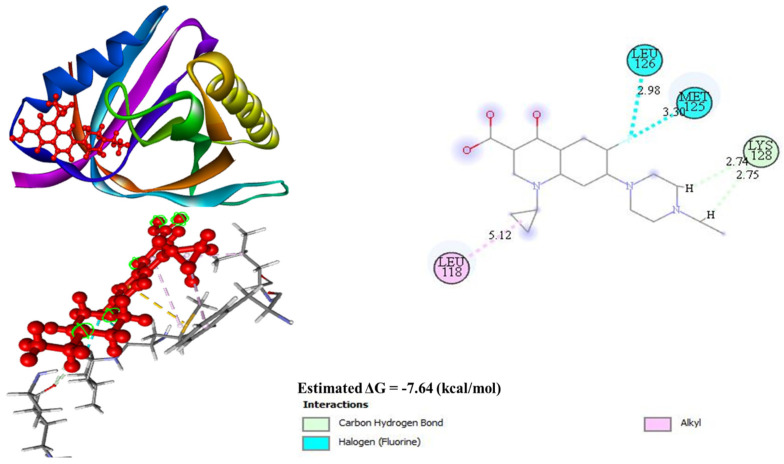
Docking results of enrofloxacin with DNA gyrase inhibitor IV at −7.64 (kcal/mol) as lowest bonding energy.

The molecular docking analysis of enrofloxacin with DNA gyrase inhibitor IV had a binding energy of −7.64 kcal/mol (Figure 8). The 2D model indicated an LYS 128 carbon–hydrogen bond with the receptor having bond lengths of 2.74Å and 2.75Å, respectively. Additionally, a halogen bond involving fluorine was identified with MET 125 and LEU 126, with a bond length of 2.98Å and 3.03Å, respectively. Other notable interactions include alkyl bonds with LEU 118 showing a bond length of 5.12Å.

**Figure 9:**
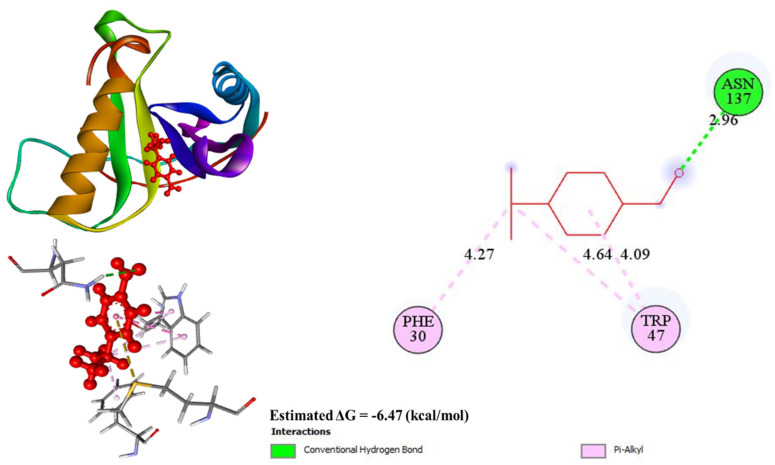
Docking results of cuminaldehyde with DNA gyrase inhibitor IV at −6.47 (kcal/mol) as lowest bonding energy.

The molecular docking analysis of cuminaldehyde with the respective proteins showed the lowest binding energy level of −6.47 Kcal/mol. Specifically, ASN 137 established a hydrogen bond with a length of 2.96Å, while two alkyl bonds were observed with TRP 47, i.e., 4.09Å and 4.64Å bond lengths, respectively. Lastly, PHE 30 was involved in an alkyl bond with a bond length of 4.27Å (Figure 9).

### 3.5. Minimum Inhibitory Concentration

**Figure 10:**
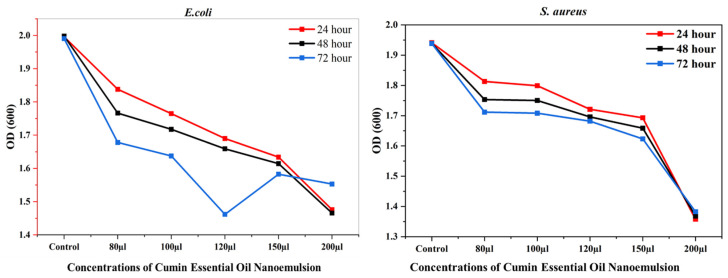
Minimum Inhibitory Concentration (MIC) of *E. coli* and *S. aureus* under different concentrations of CEONE.

The MIC for each bacterium was observed at a 200 µL concentration of CEONE. The antibacterial effect of CEONE showed significant activity at 48 and 72 h. The lowest bacterial concentration for *E. coli* was observed at 120 µL, while for *S. aureus,* it was 200 µL for CEONE at 72 h (Figure 10).

### 3.6. In Vivo Effect of CEONE on Broilers

#### 3.6.1. Growth Performance

In the first week, the growth showed no significant variation as per the BWG, FI, and FCR data shown in Table 4. During 14–42 days, no mortality was observed. The CEONE treatment showed a significant (*p* < 0.05) increase in BWG with a lower FCR compared to the positive control group. Weekly data has been presented in the Supplementary Table S6.

#### 3.6.2. Intestinal Microbiota

**Figure 11:**
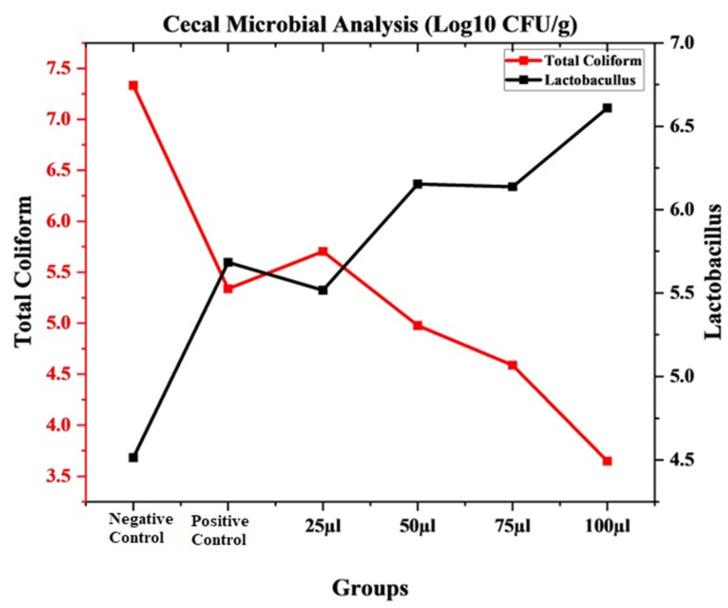
Cecal microbial count (Log 10 CFU/g).

A significant effect was observed in the mean values of *Lactobacillus* and total coliform counts when compared to the control and antibiotics treatments. A lower level of total coliform was observed with 100 µL of CEONE (Figure 11).

#### 3.6.3. Blood Hematology and Serum Biochemistry

The hematological parameters are presented in Table 5. The serum biochemistry had significant differences in HDL and LDL (Table 6).

## 4. Discussion

### 4.1. GCMS

Our experiment identified cuminaldehyde as the major antibacterial compound in CEO. This finding aligns with previous studies, which have also recognized cuminaldehyde as a significant antibacterial agent. For instance, Morteza-Semnani et al. [40] demonstrated the antibacterial efficacy of cuminaldehyde, highlighting its potential in combating various bacterial strains. Similarly, Aghababaei et al. [41] confirmed cuminaldehyde’s role as the primary antibacterial component in CEO. Homayonpour et al. [42] further supported these findings by isolating and testing cuminaldehyde, thereby establishing its strong antibacterial properties. Moreover, Xu et al. [43] provided additional evidence of cuminaldehyde’s effectiveness, reinforcing the compound’s significance in antibacterial applications. Collectively, cuminaldehyde’s position as a key antibacterial compound in CEO suggests its potential for broader antimicrobial applications.

### 4.2. Characterization of CEONE

According to Elsherif et al. [44], the droplet size of NEs is between 20 and 200 nm. Our optimization analysis agrees with other studies [42,45,46]. We agree with the observations of Alzorqi et al. [47] that the sonication interval has a significant effect on the droplets of CEONE. The small PDI exhibit a good surface area, which reflects the efficiency of NEs for further application [48]. PDI values of 0.1 to 0.25 indicate narrow distribution [49]; in our study, the PDI ranges from 0.021 to 0.1, even with a different sonication interval, cumin oil, and Tween 80 concentration, indicating a homogenous CEONE. Zeta potential, being an indicator of the NE’s physical stability, is observed in the acceptable range (higher than +30 mV or more negative than −30 mV) [49,50]. The negative zeta potential (−19.1 to −11.1) in our case indicates the sonication interval affects the stability of the NE at all sonication levels at different concentrations of cumin oil and Tween 80. The TEM analysis of nano-sized oil-loaded capsules with a clear core–shell structure confirmed the uniform dispersibility of CEONE. An inner light color of the droplet with an outer light dark spherical confirms the O/W nature of the NE. A normal distribution curve determines the size within a 20–200 nm range of the NE.

### 4.3. Drug Characterization and Oral Bioavailability

The in silico computational approach has been utilized by a variety of researchers for the validation of drug specificity with disease [29,51,52]. Swiss ADME is considered as a tool to characterize drugs for an oral availability investigation prior to their application. This process is guided by Lipinski’s Rule of Five, established in 1999, which serves as a filter to distinguish orally active drugs based on certain physicochemical properties. A threshold of 500 Dalton MW is recommended for oral drugs. Both enrofloxacin and cuminaldehyde fall within this optimal range, with cuminaldehyde exhibiting a lower MW, suggesting a potentially higher bioavailability. The balance of a compound’s solubility between octanol and water, with a Log P_o/w_ value under 5, is ideal. Enrofloxacin and cuminaldehyde possess Log P_o/w_ values of 1.64 and 2.48, respectively, aligning with the criterion. As per the Esgan filter, a WLOGP value up to 5.88 is acceptable. Here, enrofloxacin and cuminaldehyde have values of 1.91 and 2.62, indicating favorable lipophilicity. A TPSA value below 131 Å² is deemed favorable for oral drugs. Enrofloxacin and cuminaldehyde demonstrate TPSA values of 65.78 Å² and 17.07 Å², respectively, well within the recommended range [29]. Synthetic accessibility measures the ease of synthetic organic compounds’ structural complexity and the availability of precursors. This is quantified on a scale from 1 (easiest) to 10 (most difficult). In our analysis, both drugs scored well, with the organic compound having a perfect score of 1, which confirmed the synthesis. Enrofloxacin scored slightly higher at 2.73, which indicates a more complex synthesis [53]. The bioavailability score predicts a compound’s potential for achieving more than 10% oral bioavailability in rats, which corroborates with TPSA and Lipinski’s Rule of Five. An ideal score ranges from 0.25 to 6. Both drugs have a bioavailability score of 0.55, denoting a similar likelihood of oral bioavailability [54].

### 4.4. Toxicity Analysis

The toxicity analysis revealed that neither enrofloxacin nor cuminaldehyde exhibit AMES toxicity. When considering the maximum tolerated dose for humans, a threshold value of 0.477 log (mg/kg/day) is used to differentiate between low and high doses [55]. Enrofloxacin exhibited a maximum tolerated dose of 0.51 log (mg/kg/day), which is slightly above this threshold, suggesting a higher potential for adverse effects at elevated doses. In contrast, cuminaldehyde demonstrated a significantly higher tolerance level at 0.839 log (mg/kg/day), implying a greater safety margin for human consumption.

The LD_50_ value, which represents the dose lethal to 50% of a test population, is a critical measure of acute toxicity. A value below 0.5 mM indicates high toxicity. According to Amir et al. [29], LD_50_ values below 0.5 mM are considered to have a high acute toxicity. They used the same pKCSM toxicity analysis method and predicted that cuminaldehyde has a 1.7 mM LD_50_ value, which is in parallel with our prediction results using a similar tool. As part of the comparative toxicity analysis between cuminaldehyde and enrofloxacin, we found that both compounds are in the normal range, 1.7 mM (cuminaldehyde) and 2.431 mM (enrofloxacin). Additionally, neither enrofloxacin nor cuminaldehyde interfered with hERG channel functions, which is important because disturbances in these functions can lead to cardiac issues. However, hepatotoxicity was observed with enrofloxacin, indicating potential liver damage with its use. In contrast, cuminaldehyde was found to be beneficial for hepatic functions, due to the non-damaging effect on the liver. Minnow toxicity tests also yielded satisfactory results for both compounds, further supporting their safety profiles. Notably, cuminaldehyde has a higher tolerated dose compared to enrofloxacin, indicating a potentially safer margin for human use [29]. The overall findings suggest that cuminaldehyde, with its higher tolerance and beneficial hepatic effects, might be a safer alternative to enrofloxacin in certain applications.

### 4.5. Gene Functional Analysis and Molecular Docking

The sbmC gene is recognized for producing an inhibitor that targets DNA gyrase, which is an enzyme vital for introducing negative supercoils into DNA, crucial for DNA processes. The gyrase binding with DNA shields cells from toxins like microcin B17, CcdB, and synthetic quinolones and the sbmC gene’s role is to prevent alkylating agents [56]. DNA gyrase introduces negative supercoils into closed circular double-stranded DNA through an ATP-dependent mechanism, thereby preserving the chromosomes in a conformation conducive to cellular functions [57]. The ability to maintain these conformations is essential for cellular processes like replication and transcription. Furthermore, DNA gyrase facilitates the interconversion of other topological isomers of DNA, such as catenanes, and can relax negatively supercoiled DNA without the need for ATP [58]. This dual functionality makes DNA gyrase a crucial enzyme for maintaining DNA topology. Comparative studies revealed *E. coli* gyrase had a higher supercoiling activity than its complement in *M. tuberculosis* due to the faster introduction of supercoils in *E. coli* gyrase at equivalent concentrations. Moreover, the GyrB subunit from *S. typhimurium* is toxic to *E. coli*, despite a high degree of sequence similarity, underscoring the specificity of gyrase subunits within species [58,59]. This specificity in evolutionary adaptations of bacterial species is due to their environments. The differential enzymatic functions of *E. coli* gyrase and topoisomerase IV largely hinge on the C-terminal domain of GyrA, especially the GyrA-box, which is pivotal for their distinct activities [60,61]. In an ATP-independent reaction, the enzyme transiently cleaves DNA, resulting in the formation of a phosphotyrosine linkage between GyrA and the DNA [62]. Quinolones can stabilize the intermediate, which serves as a biomarker for drug toxicity [63]. The ability to be at this stable range is critical for the therapeutic and toxicological effects of quinolones.

A diverse range of inhibitors target DNA gyrase, modulating its function. These inhibitors, such as coumarins, cyclothialidines, pyrrolopyrimidines, pyrazolthiazoles, and quinolones, interact with various elements of the gyrase complex, each employing unique inhibitory pathways [64,65]. Cyclothialidines, distinct from coumarins, bind to GyrB and competitively inhibit ATPase activity, employing a unique mode of action [66,67,68,69]. This specificity highlights the potential for developing targeted antibacterial therapies. Pyrrolopyrimidines concurrently inhibit GyrB, ATPase activity, and the analogous subunit in topoisomerase 4, ParE [70]. Quinolones interact with GyrA in the DNA–enzyme complex, stabilizing a covalent reaction intermediate with the DNA [63]. Acriflavine acts to impede both DNA supercoiling and the ATPase activity induced by DNA [71,72]. QnrB4 shields DNA supercoiling from fluoroquinolone inhibition without affecting the supercoiling activity independently [73]. These findings suggest that different classes of gyrase inhibitors can be used synergistically to overcome bacterial resistance.

The comparative molecular docking with cuminaldehyde, an organic antibacterial compound, and enrofloxacin confirmed a better antibacterial activity. As cuminaldehyde is a major component of cumin and the presence of additional compounds suggests CEO could be a better substitute for synthetic antibacterial agents, this holistic approach to use natural compounds may provide a safer and more sustainable alternative to synthetic drugs, particularly in the face of rising antibiotic resistance.

### 4.6. Growth Performance

Our findings align with several studies on CEO and other related compounds on broiler growth and performance metrics. Aami-Azghadi et al. [74] reported no significant growth deviations in broilers administered CEO. However, lower concentrations of EO were associated with increased BWG during the growth phase relative to the controls. This suggests a balanced concentration of CEO can optimize growth without negatively impacting overall development. Yilmaz and Gul [75] noted an uncertain impact of CEO on broiler growth under thermal stress conditions. This uncertainty could be attributed to the complex interactions between CEO components and the stress physiology of broilers. The effectiveness of a CEO might be modulated by environmental factors, indicating the need for further studies under varying conditions.

Conversely, Kumar et al. [76] documented an increased dose of black cumin seeds stimulated BW and FI, although there was an inverse relationship with FCR. This indicates higher doses of black cumin seeds promote growth in terms of feed utilization, highlighting a trade-off between growth and feed efficiency. Torki et al. [77] explored the effects of ethanol extract of propolis (EEP) and CEO, finding that CEO had a pronounced influence on BW, BWG, and FCR. This supports that CEO can positively impact growth parameters, possibly due to its bioactive compound absorption and utilization. Guler et al. [78] investigated black cumin seed at various dosages compared to avilamycin, finding that a 1% black cumin seed concentration combined with antibiotics yielded the most significant average daily weight gain and FCR improvements compared to the control group. This synergy between black cumin seed and antibiotics suggests that CEO and its components might work best in combination with other growth-promoting agents.

Alimohamadi et al. [79] concluded that cumin seed did not significantly impact the final BW and FCR. This discrepancy might be due to differences in the experimental conditions, cumin seed preparation, or broiler breed, indicating that the effects of cumin-based treatments can be context-dependent. In alignment with our outcomes, Amiri et al. [5] observed that broilers receiving the highest concentration of nano-encapsulated cumin exhibited increased BWG, enhanced FCR, and reduced FI. These findings highlight the efficacy of nano-encapsulation in enhancing the bioavailability and potency of CEO, making it a more effective growth promoter compared to its free form, cumin extract, and cumin seeds. Collectively, these studies confirm the principle that nano-encapsulation of CEO enhances its efficacy on growth parameters. This suggests that nano-encapsulated CEO could be a viable alternative to traditional growth-promoting agents in poultry nutrition, offering improved growth performance and feed efficiency. The data underline the potential of CEONE as a best alternative to traditional growth-promoting agents in poultry nutrition.

### 4.7. Antimicrobial Activity

In vitro antibacterial analysis of nano-encapsulated CEONE demonstrated sustained release and effectiveness. This finding aligns with Hou et al. [38], who reported that the NE of CEO exhibited superior antibacterial activity compared to free CEO. This enhanced efficacy may be attributed to the improved stability and bioavailability of the NE, which allows for a more consistent and prolonged antibacterial effect. Syed et al. [80] highlighted that the bactericidal effects of nano-encapsulated EOs are comparable to their free counterparts. This similarity in efficacy allows for a reduction in the quantity of EO needed, minimizing costs and avoiding the potential for overpowering flavors in food systems. The significant antibacterial activity of CEONE, particularly against *S. aureus*, was also observed by Nirmala et al. [81], underscoring its potential as a robust antibacterial agent. Ranjbar et al. [82] found that CEONE exhibited nearly 100% antibacterial activity against *Pseudomonas aeruginosa* and 80% against *S. aureus* at a concentration of 5000 µg/mL. This high level of antibacterial activity supports the potential of CEONE as a powerful substitute for traditional antibiotics. Our study further observed a reduced total coliform count in the cecal microbial population, suggesting that CEONE can effectively reduce harmful bacterial loads in the gut. Ayoola et al. [83] found that *Nigella sativa* oil reduced the cecal coliform count in broilers, with the effect increasing with the dosage. Similarly, Fathi et al. [84] noted a decrease in cecal coliform and *E. coli* populations with the administration of 40 and 60 g/kg of black cumin seed. These findings are consistent with our results, indicating that EOs and their components can significantly impact the gut microbiota by reducing harmful bacterial counts. Khan et al. [85] observed a decrease in the cecal coliform population in broilers fed black cumin seeds, further supporting the antibacterial properties of these natural compounds.

Aghababaei et al. [41] reported that CEO, with cuminaldehyde as the main compound, exhibited significant antibacterial activity. They found that chicken fillets coated with CEO had an increased shelf life due to a reduced microbial load, including total bacterial counts, psychrotrophic bacteria, lactic acid bacteria, *Pseudomonas* spp., *Enterobacteriaceae*, *S. aureus*, yeast, and mold, on the 12th day of refrigeration (*p* < 0.05). This highlights the potential of CEO in food preservation by reducing spoilage microorganisms. Additionally, Moharreri et al. [86] observed that microencapsulated EOs had a positive effect on *Salmonella Enteritidis*-challenged broilers, significantly reducing the *S. Enteritidis* population in both the ileum and cecum (*p* < 0.05). These findings reinforce the potential of nano-encapsulation to enhance the antibacterial properties of EOs. Our research corroborates these foundational observations by revealing a reduction in the total coliform counts within the cecal microbiota. This suggests that CEONE and similar essential components can serve as viable alternatives to conventional antibiotics, offering effective antibacterial activity with potential benefits for gut health and food preservation.

### 4.8. Hematology and Serum Biochemistry

The effects of black cumin and its derivatives on blood constituents and physiological parameters have been explored in several studies. Laudadio et al. [87] found black cumin had no significant effect on blood constituents. This discrepancy could be due to differences in the dosage, experimental conditions, or the specific strain of black cumin used. In contrast, Fathi et al. [84] observed significant effects of black cumin, including increased levels of RBCs, Hb, and HCT, along with reduced levels of ALT, total cholesterol, and triglycerides. These findings suggest black cumin has a beneficial impact on hematological parameters and lipid metabolism, due to its bioactive compounds with antioxidant properties. They scavenge free radicals and prevent lipid peroxidation. Lipid oxidation contributes to dyslipidemia and metabolic disorders. By inhibiting lipid peroxidation, CEO helps to maintain lipid homeostasis in broilers [88,89]. Some CEO compounds (e.g., thymol) activate peroxisome proliferator-activated receptor gamma coactivator 1-alpha (PGC-1α). PGC-1α regulates mitochondrial biogenesis and thermogenesis. Increased thermogenesis enhances energy expenditure, potentially influencing lipid utilization and preventing excessive fat accumulation [90]. A balanced gut microbiome also affects lipid absorption, metabolism, and inflammation. By promoting beneficial bacteria and suppressing harmful ones, the CEO could indirectly impact lipid homeostasis [91]. NE’s form increases the bioavailability of the active compounds for the better absorption and utilization of active compounds. Amiri et al. [5] reported that nano-encapsulated CEO decreased the levels of cholesterol, triglycerides, and LDL while improving FCR. The nano-encapsulation likely enhances the bioavailability and efficacy of CEO, resulting in more pronounced physiological benefits. Khan et al. [85] noted an increase in the total protein and hematological parameters with higher concentrations of black cumin seed, indicating a dose-dependent effect on protein synthesis and blood health. This supports the potential of black cumin to improve the overall health and nutritional status in broilers.

Aydogan et al. [92] observed no significant effect of black cumin compared to garlic, suggesting that the specific bioactive compounds and their concentrations in black cumin and garlic may influence their comparative effectiveness. This highlights the importance of considering the unique properties of different herbal supplements. Aami-Azghadi et al. [74] found a non-significant effect on broiler physiology but observed an improved final BW. This suggests that while black cumin might not directly affect the physiological parameters, it could still promote growth, possibly through improved nutrient utilization or immune modulation. Al-Beitawi and El-Ghousein [93] conducted studies like ours, showing that *Nigella sativa* increased HDL and decreased LDL in the treated groups compared to the control groups. This aligns with our findings and suggests that black cumin and its derivatives may improve lipid profiles, contributing to better cardiovascular health. Alimohamadi et al. [79] compared the effects of black seed and cumin in broilers, noting that black seed increased the RBC count, Hb concentration, and HCT percentage compared to the control diet, with a significant decrease in total cholesterol and LDL. These results suggest black seed may have more potent hematological and lipid-lowering effects compared to cumin, possibly due to differences in their phytochemical compositions. The black cumin and its nano-encapsulated forms positively influenced the blood lipid profiles. The improved bioavailability of nano-encapsulated CEO underscores its potential as an effective supplement for enhancing broiler health and performance.

## 5. Conclusions

This study systematically explored the potential of CEONE as a multifaceted agent, integrating chemical, physical, biological, and computational perspectives. The GCMS analysis identified cuminaldehyde as the predominant antibacterial compound in CEO, corroborating its well-established antimicrobial efficacy. Characterization of CEONE revealed optimized NE properties, including a small droplet size (85 nm), lower PDI, and negative zeta potential, indicative of a stable and homogeneously dispersed NE suitable for various applications. In silico analysis using Swiss ADME highlighted the favorable drug-like properties for both cuminaldehyde and enrofloxacin, underscoring their potential as oral bioavailable agents. Toxicity assessments indicated the safety of cuminaldehyde, contrasting with the hepatotoxic effects observed with enrofloxacin, suggesting a safer alternative for certain applications. Molecular docking simulations elucidated interactions between cuminaldehyde and bacterial DNA gyrase that suggest potential mechanisms of antibacterial action comparable to synthetic counterparts like enrofloxacin. Experimental validation through MIC assays confirmed CEONE’s significant antibacterial activity against *E. coli* and *S. aureus*, reinforcing its therapeutic potential. Furthermore, in vivo studies on broilers demonstrated CEONE’s positive impact on growth performance and intestinal microbiota, highlighting its potential as a growth promoter and gut health enhancer in poultry farming. The observed effects on blood hematology and serum biochemistry attributes further underscore CEONE’s safety and potential utility in animal husbandry. Overall, CEONE emerges as a promising candidate for diverse applications in pharmaceuticals, agriculture, and food industries, offering a natural, effective, and safe alternative to synthetic antimicrobial agents. Future research should focus on scaling up production and conducting clinical trials to validate its efficacy and safety in human applications.

## Figures and Tables

**Table 1:** Basal diet composition (g/kg). The numbers in brackets are days.

Item	Starter (0–10)	Grower (11–24)	Finisher (25–42)
Maize	63	65	61.5
Soya	2.5	2	3
Gluton 30%	1	-	-
Sunflower seeds	5	-	4%
Fish meal	8	11	10
Canola	-	-	1
Rice polish	10	10	10
Rape seed	2	3	2
Wheat bran	5	5	5
Calcium carbonate	0.5	0.5	0.4
Choline	0.1	0.1	0.1
Lysine	1	0.86	0.82
Threonine	0.22	0.19	0.17
Arginine	0.37	0.35	0.27
Tryptophan	0.04	0.05	0.039
Valine	0.20	0.18	0.15
DL-methionine	0.22	0.19	0.26
Isoleucine	0.26	0.22	0.22
Vitamin premix	0.25	0.25	0.25

**Table 2:** Physicochemical properties of drugs.

Model Name	MW (g/mol)	Heavy Atoms	Aromatic Heavy Atoms	Fraction Csp^3^	Rotatable Bonds	H-Bond Acceptors	Molar Refractivity	TPSA (Å²)
Enrofloxacin	359.39	26	10	0.47	5	5	104.95	65.78
Cuminaldehyde	148.2	11	6	0.3	2	1	46.41	17.07

MW = molecular weight, TPSA= total polar surface area.

**Table 3:** pkCSM server toxicity prediction. (https://biosig.lab.uq.edu.au/pkcsm/prediction, accessed on 20 March 2024) ^1^.

Parameters	Unit	Enrofloxacin	Cuminaldehyde
AMES ^2^ toxicity	Yes/No	No	No
Max. tolerated dose (human)	(log mg/kg/day)	0.51	0.839
hERG I inhibitor	Yes/No	No	No
hERG II inhibitor	Yes/No	No	No
Oral rat acute toxicity (LD_50_) ^3^	mol/kg	2.431	1.7
Oral rat chronic toxicity (LOAEL ^4^)	log mg/kg BW/day	1.891	2.194
Hepatotoxicity	Yes/No	Yes	No
Skin sensitization	Yes/No	No	Yes
*T. pyriformis* toxicity ^5^	log µg/L	0.288	0.766
Minnow toxicity	log mM	2.408	0.819

^1^ hERG, also known as human ether-a-go-go-related gene, encodes the potassium channel Kv11; AMES toxicity: used to assess the potential carcinogenic effect of chemicals by using the bacterial strain *Salmonella typhimurium*. ^2^ AMES: salmonella/microsome mutagenicity assay (salmonella test; Ames test). ^3^ LD_50_: lethal dose 50. ^4^ LOAEL: lowest observed adverse effect level. ^5^ *T. pyriformis* toxicity: tetrahymena pyriformis.

**Table 4:** Effect of CEONE overall growth performance of broilers.

	NC	PC	25 µL	50 µL	75 µL	100 µL	*p*-Value
BWG	976.47 ± 11.82 ^b^	1078.81 ± 10.21 ^ab^	1054.10 ± 24.34 ^ab^	1108.55 ± 21.61 ^a^	1071.07 ± 32.15 ^ab^	1116.22 ± 29.04 ^a^	0.01
FI	2223.01 ± 44.21	2301.39 ± 22.01	2208.45 ± 9.22	2291.89 ± 40.43	2325.89 ± 21.79	2284.04 ± 34.60	0.12
FCR	2.27 ± 0.02 ^a^	2.13 ± 0.00 ^ab^	2.09 ± 0.04 ^ab^	2.06 ± 0.06 ^ab^	2.17 ± 0.06 ^b^	2.04 ± 0.02 ^b^	0.02

Negative control (NC) = basal diet, positive control (PC) = enorfloxacine + basal diet; 25 µL = 25 µL NE (NE: nanoemulsion) + basal diet; 50 µL = 50 µL NE + basal diet; 75 µL = 75 µL NE + basal diet; 100 µL = 100 µL NE + basal diet. ^ab^ Means in each row with different superscripts are statistically different (*p* < 0.05).

**Table 5:** Effect of CEONE on blood hematology of broilers.

	NC	PC	25 µL	50 µL	75 µL	100 µL	*p*-Value
RBC (10^6^/µL)	2.33 ± 0.19	2.31 ± 0.25	2.09 ± 0.13	2.32 ± 0.21	2.59 ± 0.16	2.61 ± 0.22	0.48
WBC (10^3^/µL)	22.59 ± 0.34	21.98 ± 0.18	22.58 ± 0.25	22.13 ± 0.15	22.36 ± 0.12	21.73 ± 0.05	0.06
HB (g/dl)	12.64 ± 0.15	12.46 ± 0.21	12.32 ± 0.17	12.48 ± 0.15	12.77 ± 0.19	12.50 ± 0.19	0.63
LYM %	50.95 ± 0.19	50.76 ± 0.18	52.56 ± 0.16	53.16 ± 0.55	53.18 ± 0.53	53.26 ± 0.25	0.09
PLT	101.96 ± 0.22	101.97 ± 0.19	102.90 ± 0.18	103.87 ± 0.18	102.08 ± 0.16	104.54 ± 1.3	0.08

Negative control (NC) = basal diet, positive control (PC) = enorfloxacine + basal diet; 25 µL = 25 µL NE + basal diet; 50 µL = 50 µL NE + basal diet; 75 µL = 75 µL NE + basal diet; 100 µL = 100 µL NE + basal diet.

**Table 6:** Effect of CEONE on serum biochemistry parameters.

	NC	PC	25 µL	50 µL	75 µL	100 µL	*p*-Value
ALT (U/L)	71.07 ± 2.92	73.66 ± 2.33	69.91 ± 3.43	66.15 ± 4.50	68.49 ± 4.38	69.41 ± 4.29	0.80
AST (U/L)	122.12 ± 0.72	123.72 ± 1.38	125.74 ± 2.90	126.67 ± 1.10	124.52 ± 1.04	125.24 ± 2.51	0.57
HDL (mg/dL)	73.44 ± 0.80 ^b^	78.69 ± 0.66 ^a^	75.29 ± 1.04 ^ab^	76.41 ± 1.51 ^ab^	74.51 ± 1.23 ^ab^	77.87 ± 0.74 ^ab^	0.03
LDL (mg/dL)	65.54 ± 1.86 ^a^	54.66 ± 5.23 ^ab^	63.85 ± 3.35 ^ab^	55.66 ± 1.36 ^ab^	52.05 ± 1.91 ^ab^	49.99 ± 3.11 ^b^	0.01
Total protein (g/dL)	3.05 ± 0.02	3.57 ± 0.19	3.56 ± 0.39	3.21 ± 0.16	3.59 ± 0.20	3.32 ± 0.12	0.41
Triglycerides (mg/dL)	83.45 ± 0.16	85.66 ± 2.33	82.98 ± 1.70	81.98 ± 1.70	76.28 ± 3.83	81.88 ± 1.76	0.14

Negative control (NC) = basal diet, positive control (PC) = enorfloxacine + basal diet; 25 µL = 25 µL NE + basal diet; 50 µL = 50 µL NE + basal diet; 75 µL = 75 µL NE + basal diet; 100 µL = 100 µL NE + basal diet. ^ab^ Means in each row with different superscripts are statistically different (*p* < 0.05).

## Data Availability

All the relevant required datasets can be obtained from the corresponding author on special request.

## Supplementary Materials

The following supporting information can be downloaded at: https://www.mdpi.com/article/10.3390/ani14192860/s1, Figure S1: Protein validation by Ramachandran Plot using PROCHECK analysis; Table S1: Water solubility of drugs; Table S2: Drugs Likeness; Table S3: Lipophilicity of Drugs; Table S4: Medicinal Chemistry of Drugs; Table S5: Medicinal Chemistry of Drugs; Table S6: Weekly growth performance data of CEONE.
