# Supplementary material for: Preparation and Characterization of Cumin Essential Oil Nanoemulsion (CEONE) as an Antibacterial Agent and Growth Promoter in Broilers: A Study on Efficacy, Safety, and Health Impact
Source: Animals (Basel). 2024 Oct 4;14(19):2860. doi: 10.3390/ani14192860 (PMC11475229; doi:10.3390/ani14192860)
Supplement: Supplementary file 1 [file animals-14-02860-s001.zip › animals-3114037-supplementary.pdf]

Supplementary Tables

S1: Water solubility of drugs

Model Name	Log S (ESOL)	Class (ESOL)	Log S (Ali)	Class (Ali)	Log S (SILICOS-IT)	Class (SILICOS-IT)
Enrofloxacin	-1.93	Very soluble	-0.67	Very soluble	-3.56	Soluble
Cuminaldehyde	-2.52	Soluble	-2.37	Soluble	-3.15	Soluble

S2: Drugs Likeness

Model Name	Lipinski	Ghose	Veber	Egan	Muegge	Bioavailability Score
Enrofloxacin	Yes	Yes	Yes	Yes	Yes	0.55
Cuminaldehyde	Yes	Yes	Yes	Yes	Yes	0.55

S3: Lipophilicity of Drugs

Model Name	Log Po/w (iLOGP)	Log Po/w XLOGP3	Log Po/w WLOGP	Log Po/w MLOGP	Log Po/w (Silicos-IT)	Consensus Log $P_{o/w}$
Enrofloxacin	2.58	-0.25	1.91	1.75	2.21	1.64
Cuminaldehyde	2.03	2.37	2.62	2.4	2.96	2.48

S4: Medicinal Chemistry of Drugs

Model Name	Pains	Brenk	Lead likeness	Synthetic Accessibility
Enrofloxacin	0	0	1	2.73
Cuminaldehyde	0	1	1	1

S5: Medicinal Chemistry of Drugs

Model Name	GI absorption	BBB	P-gp substrate	CYP1A2 inhibitor	CYP2C19 inhibitor	CYP2C9 inhibitor	CYP2D6 inhibitor	CYP3A4 inhibitor	Skin Permeability (cm/s)
Enrofloxacin	High	Yes	Yes	No	No	No	Yes	No	-8.67
Cuminaldehyde	High	Yes	No	Yes	No	No	No	No	-5.52

BBB = blood-brain barrier, GI = gastrointestinal, CYP = cytochrome, log Kp = skin permeation, Pgp = P glycoprotein.

S6: Weekly growth performance data of CEONE

	**NC**	**PC**	**25µl**	**50µl**	**75µl**	**100µl**	***P-Value***
				**Day 15**			
BW (g)	281.55±36.08	295.34±20.36	291.07±17.79	288.06±18.64	275.03±27.41	291.31±28.11	0.99
				**Day 21**			
BW (g)	544.04±38.02	549.55±38.19	584.50±7.37	566.06±38.89	567.10±37.04	576.00±1.83	0.93
BWG (g)	262.49±29.79	254.21±58.55	293.43±10.62	277.99±26.14	292.07±51.98	284.68±29.58	0.96
FI (g)	488.40±15.47	522.87±12.29	485.79±15.00	533.87±34.62	529.30±31.15	495.29±13.12	0.47
FCR (g:g)	1.90±0.19	2.34±0.64	1.66±0.10	1.97±0.29	1.93±0.35	1.77±0.15	0.79
				**Day 28**			
BW (g)	881.62±32.90	999.88±3.02	1015.74±57.81	998.59±33.41	1001.09±48.65	998.92±15.68	0.18
BWG (g)	337.58±10.68	450.33±35.29	431.23±60.47	432.52±56.71	433.98±61.32	422.92±14.51	0.56
FI (g)	762.62±25.18	785.60±10.05	757.09±21.58	755.38±24.64	788.25±8.47	780.28±21.11	0.72
FCR (g:g)	2.26±0.13	1.76±0.15	1.82±0.23	1.81±0.28	1.88±0.25	1.84±0.06	0.55
				**Day 42**			
BW (g)	1258.03±46.32	1374.15±16.40	1345.18±42.14	1396.62±20.51	1346.11±58.30	1407.53±21.05	0.14
BWG (g)	376.40±38.06	374.27±18.56	329.44±75.85	398.03±52.66	345.01±28.41	408.60±9.08	0.77
FI (g)	971.98±18.33	992.91±8.92	965.57±19.60	1002.63±16.38	1008.34±8.48	1008.46±7.02	0.19
FCR (g:g)	2.62±0.21	2.66±0.12	3.23±0.68	2.60±0.33	2.96±0.23	2.47±0.04	0.64
				**Overall 15-42**			
BWG	976.47±11.82^{b}	1078.81±10.21^{ab}	1054.10±24.34^{ab}	1108.55±21.61^{a}	1071.07±32.15^{ab}	1116.22±29.04^{a}	0.01
FI	2223.01±44.21	2301.39±22.01	2208.45±9.22	2291.89±40.43	2325.89±21.79	2284.04±34.60	0.12
FCR	2.27±0.02^{a}	2.13±0.00^{ab}	2.09±0.04^{ab}	2.06±0.06^{ab}	2.17±0.06^{b}	2.04±0.02^{b}	0.02

Negative control (NC) = basal diet, positive control (PC) = enorfloxacine + basal diet; 25 µL = 25 µL NE (NE: nanoemulsion) + basal diet; 50 µL = 50 µL NE + basal diet; 75 µL = 75 µL NE + basal diet; 100 µL = 100 µL NE + basal diet. $^{(ab)}$Means in each row with different superscripts are sta-tistically different ($p < 0.05$).

Supplementary Figure

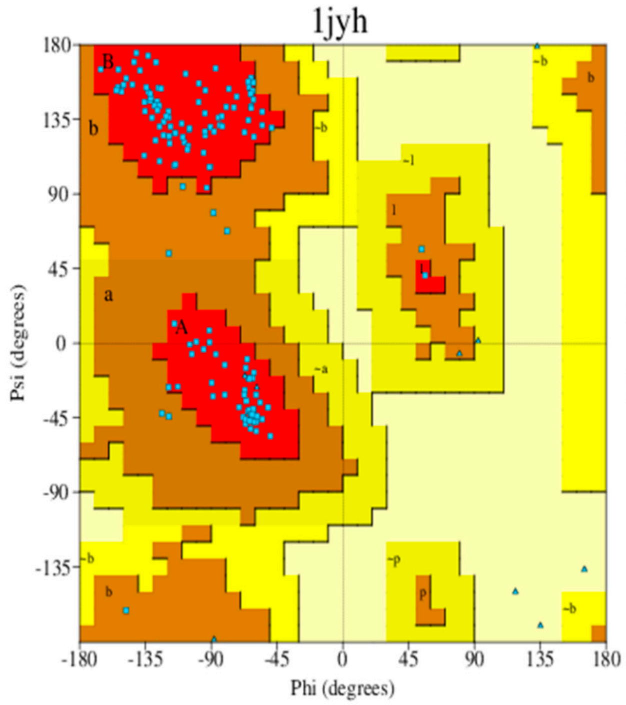

Plot statistics

Residues in most favoured regions [A,B,L]	125	94.0%
Residues in additional allowed regions [a,b,l,p]	8	6.0%
Residues in generously allowed regions [~a,~b,~l,~p]	0	0.0%
Residues in disallowed regions	0	0.0%
	----	------
Number of non-glycine and non-proline residues	133	100.0%
Number of end-residues (excl. Gly and Pro)	2	
Number of glycine residues (shown as triangles)	10	
Number of proline residues	10	

Total number of residues	155	

Based on an analysis of 118 structures of resolution of at least 2.0 Angstroms and R-factor no greater than 20%, a good quality model would be expected to have over 90% in the most favoured regions.

S1: Protein validation by Ramachandran Plot using PROCHECK analysis.
